# The timing of perinatal hypoxia/ischemia events in term neonates: a retrospective autopsy study. HSPs, ORP-150 and COX2 are reliable markers to classify acute, perinatal events

**DOI:** 10.1186/1746-1596-5-49

**Published:** 2010-07-13

**Authors:** Irene Riezzo, Margherita Neri, Francesco De Stefano, Ezio Fulcheri, Francesco Ventura, Cristoforo Pomara, Roberto Rabozzi, Emanuela Turillazzi, Vittorio Fineschi

**Affiliations:** 1 From the Department of Forensic Pathology, University of Foggia, Foggia, Italy; 2 From the Department of Forensic Medicine, University of Genoa, Genoa, Italy; 3 From the Department of Pathology, University of Genoa, Genoa, Italy

## Abstract

**Background:**

The understanding of the cellular responses implicated in perinatal brain damages and the characterization of the various mechanisms involved might open new horizons for understanding the time of onset of a brain hypoxic-ischemic lesion and for effective therapeutic strategies.

**Methods:**

We performed an immunohistochemical investigation on brain and brainstem sections of 47 peripartum deaths. The gradation and localization of the expression of antibodies such as TNFα, IL-1β, IL-6, HSPs, β APP, anti-TrypH, GAP43, GFAP, COX2, ORP-150, could be correlated with an hypoxic-ischemic damage to document a significant correlation between response and the time of onset acute (≤8 hs) or non-acute (≥8 hs ≤48 hs).

**Results and Discussions:**

In non-acute cases HSP70 reaction was prominent in the neuron cytoplasm, while in acute cases a mild reaction was evident in sporadic fields. HSP90 exhibited a similar pattern of positivity as HSP70. In acute group, ORP150 expressed an intense reaction showing a granular pattern in the cytoplasm of the neurons in the cortex of the infarcted areas. In non-acute group the positive reaction was more intense in astrocytes and less extended in neurons. COX2 reaction exhibited the strongest positive reaction in the neuronal cell bodies of acute cases, while a immunolabeling was prominent in the glial cytoplasm in the non-acute cases.

**Conclusions:**

Chaperones HSP70 and 90, ORP-150 reaction, and COX2 protein, have provided very interesting results. These results would suggest to the clinicians to extend the differential diagnosis of a too large perinatal hypoxic-ischemic insult category to delineate a more accurate chronological judgement.

## Introduction

The timing of perinatal hypoxia is complex and incompletely understood. It is traditionally based on clinical, laboratory and instrumental criteria which are nonspecific markers of a difficult birth [1,2]. These non-specific intra-partum markers provide poor information on the timing and duration of an asphyxiating insult [3]. Autopsy, placental and cord examination, laboratory tests and genetic studies may explain both the cause of death and the time of onset of the neuropathology. Brain histological examination can provide useful information on the timing of an hypoxic - ischemic lesion; the patterns of perinatal brain injury depend on the aetiology and the stage of development of the foetal nervous system, since the vulnerabilities of gray and white matter differ depending on post-conceptional age and on neuro-anatomic site [4,5]. New insights into the mechanisms involved in neonatal hypoxic-ischemic brain injury have recently transformed the old concept that most cases are the results of an acute hypoxia during labour and delivery. Current knowledge on the chronology of the response of cerebral tissue following the occurrence of a hypoxic insult emphasizes immunohistochemical investigations on brain specimens as useful tools in perinatal-related death autopsy. Understanding the time of onset of the brain lesion is of paramount importance to medical and legal professionals. In Courts, in fact, much of the debate focuses on whether or not there is evidence of acute intrapartum hypoxia and if so, whether the care provided was timely and adequate [6,7].

In the present study we performed an immunohistochemical investigation to detect brain markers expressed during the perinatal hypoxic-ischemic event and to determine if and to which extent, the gradation of the expression of markers such as Tumour Necrosis Factor-alfa (TNFα), Interleukins (IL-1β, IL-6), macrophage marker (CD68), Heat Shock Proteins (HSPs), β Amyloid Precursor Protein (β APP), anti-Ttryptophan Hydroxylase (anti-TrypH), Growth Associated Protein43 (GAP43), Glial Fibrillar Acidic Protein (GFAP), Cyclooxygenase2 (COX2), Oxygen-Regulated Protein150 (ORP-150), could be correlated with an hypoxic-ischemic damage to document a significant correlation between response and time of onset (acute or non-acute).

## Materials and methods

We proceeded to review the autopsies performed on newborns who had died following a difficult birth at the Departments of Forensic Pathology of the University of Foggia and at the University of Genoa during the period 1999-2008, for a total of 47 peripartum deaths.

Peripartum cases were represented by newborns with a gestational age between 40 and 42 weeks dying within 48 hours after birth. Post-mortem examination was performed within 24 hours from death. We excluded all cases in which congenital anomalies and/or central nervous system or cardiac malformations, infections, chromosomal or metabolic abnormalities were diagnosed.

On the basis of clinical data (pregnancy, labour, fetal cardiotocography (CTG) tracing and Apgar score) the cases were so divided:

▪ Cases in which the hypoxic insult has acted in a non-acute manner (≥8 hs ≤48 hs) during the late period of pregnancy: n = 23 (non-acute group);

▪ Cases in which the hypoxic-ischemic insult has occurred in an acute (≤8 hs) and sudden way during the labour: n = 24 (acute group);

▪ Perinatal death (trauma and infanticides) with immediate neonatal death due to brain traumatic injuries: n = 15 (control group).

Significant macroscopic findings were searched for distinctive patterns of damage, specifically in the deep gray and underlying white matters of the midbrain, lateral geniculate bodies, posterior putamen and peri-rolandic cortex. We looked for principal types of acute haemorrhagic and hypoxic-ischaemic brain damage in the neonate such as

▪ Haemorrhagic lesions (germinal matrix and intraventricular haemorrhage, choroid plexus haemorrhage, cerebellar haemorrhage, subpial haemorrhage);

▪ White matter destructive lesions such as periventricular leukomalacia, subcortical leukomalacia (with or without infarction of overlying cerebral cortex), telencephalic leukomalacia (selective loss of myelination glia);

▪ Grey matter destructive lesions such as pontosubicular necrosis, acute degeneration of other neuronal groups and infarction especially in the cerebral cortex, basal ganglia, thalamus, brain stem [5].

Standard sample blocks were taken from the cerebral cortex, white and grey matters, basal ganglia, thalami and the brainstem. All tissue samples were fixed in 10% formalin for 48 hours and then processed and embedded in paraffin. For each case, total sections of about 4 μm thickness were cut and stained with haematoxylin and eosin (H&E), Golgi, Perls, and Von Kossa methods.

An immunohistochemical study was performed on tissue sections using a panel of antibodies chosen on the basis of the most recent scientific literature on the matter of brain hypoxic-ischemic damage: GFAP, TNFα, IL-1β, IL-6, CD68 (MAC387 clone), HSP27, 70 and 90, COX2, ORP150, β-APP, TrypH, GAP-43, apoptosis (TUNEL). The sections in paraffin were rehydrated and incubated for 20 min in methanol containing 10% of H_2_O_2 _to block endogenous peroxidase. The sections were pretreated to facilitate antigen retrieval and to increase membrane permeability to antibodies and were then incubated with the primary antibody (see Table 1). The detection system utilized was the LSAB+ kit (Dako, Copenhagen, Denmark), a refined avidin-biotin technique in which a biotinylated secondary antibody reacts with several peroxidase-conjugated streptavidin molecules. The positive reaction was visualized by 3,3-diaminobenzidine (DAB) peroxidation according to standard methods. The sections were counterstained with Mayer's haematoxylin, dehydrated, coverslipped, and observed in a Leica DM4000B optical microscope (Leica, Germany) connected to a computerized system with a photo camera (DC 480 Leica, Germany). The samples were also examined under a confocal laser scanning microscope and a three-dimensional reconstruction was performed (True Confocal Scanner TCS SPE, Leica, Germany). A preliminary semi-quantitative evaluation of the immunohistochemical findings was made by two different investigators without prior knowledge.

**Table 1:** Panel of antibodies selected in the study

Antibody against	Pre--treatment	Incubation time of primary antibody, temperature	Concentration of primary antibody
GFAP (Dako, Copenhagen, Denmark)	No pre-treatment	120 min, 20°C	1:300

TNFα (Santa Cruz, CA, USA)	boiling in 0.1 M Citric Acid buffer.	120 min, 20°C	1:600

IL-1β (Santa Cruz, CA, USA)	boiling in 0.25 mM EDTA buffer.	120 min, 20°C	1:4000

IL-6 (Santa Cruz, CA, USA)	5 min Proteolytic Enzyme (Dako, Copenhagen, Denmark), 20°C.	120 min, 20°C	1:2000

CD68 (Serotec, United Kingdom)	5 min Proteolytic Enzyme (Dako, Copenhagen, Denmark), 20°C.	120 min, 20°C	1:200

HSP 27 (NovaCastra, United Kingdom)	boiling in 0.25 mM EDTA buffer..	120 min, 20°C	1:20

HSP 70 (NovaCastra, United Kingdom)	boiling in 0.25 mM EDTA buffer..	Overnight, 20°C	1:100

HSP 90 (NovaCastra, United Kingdom)	boiling in 0.25 mM EDTA buffer..	120 min, 20°C	1:50

COX2 (Santa Cruz, CA, USA)	5 min Proteolytic Enzyme (Dako, Copenhagen, Denmark), 20°C.	120 min, 20°C	1:100

ORP-150 (IBL, Gunma, Japan)	boiling in 0.1 M Citric Acid buffer.	120 min, 20°C	1:200

β-APP (NovaCastra, United Kingdom)	boiling in 0.1 M Citric Acid buffer.	Overnight, 20°C	1:300

TrypH (NovaCastra, United Kingdom)	boiling in 0.1 M Citric Acid buffer.	Overnight, 20°C	1:50

GAP-43 (Santa Cruz, CA, USA)	boiling in 0.1 M Citric Acid buffer	120 min, 20°C	1:2000

TdT enzyme (Chemicon, CA, USA)	15 min in Proteinase K (20 μg/ml), 20°C	60 min, 38°C	30 μl of TdT in 70 μl of reaction buffer (Chemicon, CA, USA)

The reactions were graded as follows: 1. (0): not expressed; 2. (+): isolated and disseminated expression; 3. (++): expression in groups or widespread foci; 4. (+++): widespread expression.

### Statistical Analysis

Semi-quantitative evaluation of the immunohistochemical findings and gradation of the immunohistochemical reaction were described with an ordinal scale and the median value reported. Analysis of variance for the non parametric data between groups was performed using Kruskal-Wallis test. When differences were found to be significant, analysis between the unmatched groups were performed with a Dunn's Multiple Comparison post hoc test. Significance level was set to 5% (SPSS ver. 16.01 for Windows - SPSS Inc., Chicago USA)

## Results

**Figure 1:**
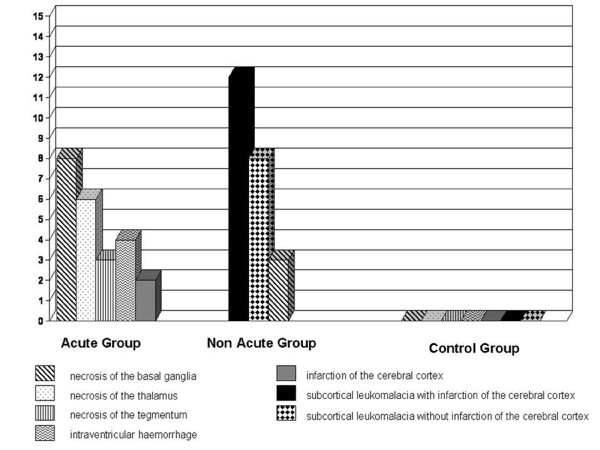
**Pathological findings in acute and non-acute groups**.

**Figure 2:**
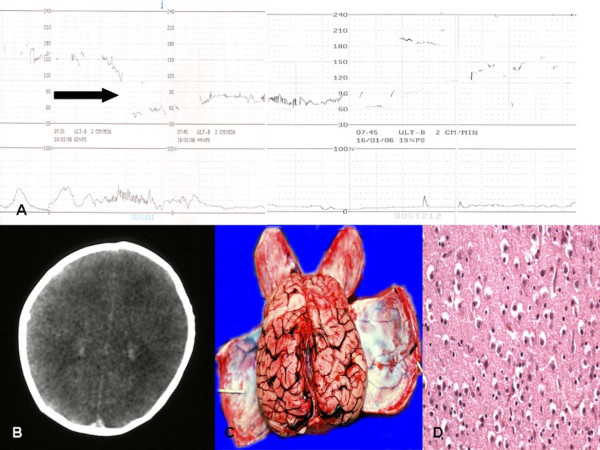
**(A) CTG tracing**. Sudden decelerations (arrow) that do not recover: urgent delivery ("rescue") is indicated. This tracing permits the diagnosis that the fetus (40ws.) suffered an acute, rather profound, ischemic event just prior to delivery due to the mother's prolonged cardiac arrest. **(B-C) **Same case as in (A): CT (midventricular level) appearance with enlarged lateral ventricles and generalized brain swelling at autopsy. **(D) **Histology of the cortical layer shows it to be oedematous with nuclear pyknosis.

The pathological findings are summarized in Figure 1. In the acute group necrosis of the basal ganglia (8 cases) and of the thalamus (6 cases) were observed; intraventricular haemorrhages (4 cases), necrosis of tegmentum (3 cases) and two cases of infarction of the cerebral cortex were described. Three cases of necrosis of the basal ganglia, 12 cases of subcortical leukomalacia with infarction of the cerebral cortex and 8 cases without infarction were observed in the non-acute group. In acute insult the microscopic study of the brain samples, typically showed massive brain oedema (Figure 2), bleeding into the choroid plexus with massive intra-ventricular haemorrhages, sub-pial haemorrhages involving the cerebrum or cerebellum. Neuronal apoptosis and necrosis were observed. Frank infarction affected the cerebral cortex and white matter, in which glial cells had vesicular nuclei and fine fibrillary cytoplasmic processes. Necrosis was observed in the basal ganglia, thalamus, internal granular layer of the cerebellar cortex, inferior colliculi, inferior olivary nucleus and brainstem tegmentum.

**Figure 3:**
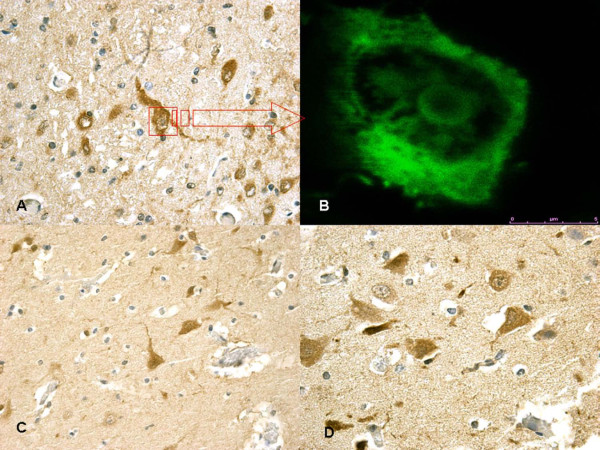
**(A) In non-acute cases (~10 hs) HSP70 reaction was prominent in the neuron cytoplasm of the affected regions**. **(B) **(insert) Confocal laser scanning microscope: typical morphological features of neuronal (green) apoptosis associated with marked condensation of chromatin and its fragmentation into discrete bodies. **(C-D) **In acute cases a mild HSP70 reaction was evident in sporadic fields.

**Figure 4:**
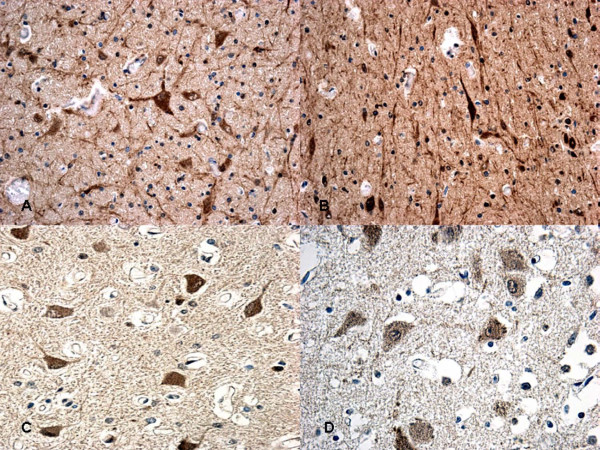
**Immunohistochemical staining for HSP90 at 12 hs (A-B) and at 15 hs (C) exhibited an intense reaction in the cytoplasm of the neurons**. The reaction must be compared with acute case (≤8 hs) **(D) **in which a mildly granular reaction is evident in the neurons.

**Figure 5:**
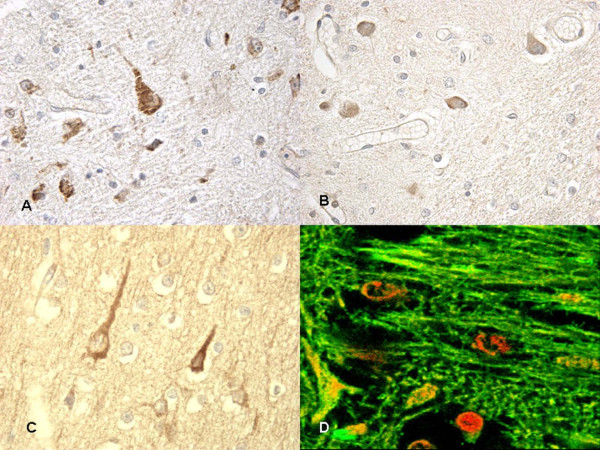
**ORP150 immunohistochemical expression related to cell viability: (A-B) in non-acute case we detected mildly neuron reaction and positive astrocytes stain**. **(C) **Strong neuron reaction to ORP150 in acute case. **(D) **Confocal laser scanning microscope: cortical layers showed intense and diffuse reactions granular pattern in the cytoplasm of the neurons (ORP150 reaction is in orange).

**Figure 6:**
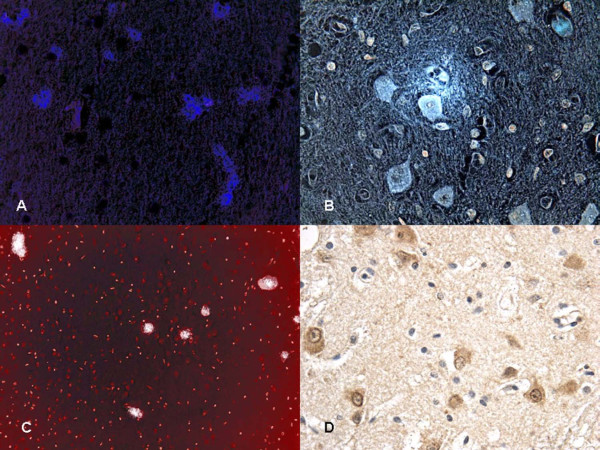
**Confocal laser scanning microscope: COX2 positivity at (A) vascular level demonstrated in (C) too**. **(B) **COX2 immunolabeling was prominent in the glial cytoplasm in the non-acute cases while **(D) **COX2 reaction exhibited a strong positive reaction in the neuronal cell bodies in the acute cases.

Particularly significant was the immunohistochemical study of the brain and brainstem sections. In non-acute cases HSP70 reaction was prominent in the neuron cytoplasm of the affected regions, while in acute cases a mild reaction was evident in sporadic fields (Figure 3). HSP90 exhibited a similar pattern of positivity as HSP70 (Figure 4). In the acute group, ORP150 expressed an intense reaction showing a granular pattern in the cytoplasm of the neurons in the cortex of the infarcted areas. Differently, in the non-acute group the positive reaction was more intense in astrocytes and less extended in neurons (Figure 5). COX2 reaction exhibited a strong positive reaction in the neuronal cell bodies while immunolabeling was prominent in the glial cytoplasm in the non-acute cases (Figure 6).

The reaction with GFAP has shown a constant and strong positivity in astrocytes, which was documented also in control cases, without significant differences in the different groups, therefore not allowing to distinguish them. However, the reaction represents an excellent morphological definition of astrocytes, therefore permitting to recognise the different kinds of astrocytes which express GFAP in different ways, so as to better identify the cells where the positive reaction is a consequence of a hypoxic-ischemic insult.

The reaction to β-APP has shown a strong positivity not only in the axons but also in the neuronal body and in the glia in control cases. In the non-acute cases the intensity of neuronal and glial expression has shown an almost constant and widespread positivity, therefore confirming data reported in literature showing a positive expression of the protein already during acute stages (<3 hours) [8].

TrypH and GAP-43 did not present significant results. Reaction to TrypH was weakly positive mainly in neurons especially in some cases belonging to the acute group, while the reaction to GAP-43 showed positivity limited exclusively to glial cells in a few non-acute cases and only vascular in some acute cases.

Regarding cytokines (IL-1β, IL-6, TNFα), we observed a mild and sporadic positive reaction in neurons only in some non-acute cases (25%), associated to vascular and glial positivity. The expression in acute cases was shown only at vascular level. In particular, the reaction to IL-1 β, although weak, was positive mainly in neurons since the acute stages and in neurons but also in glial cells in the following stage (non-acute).

CD68, marker of microglia, did not present conclusive results, since sporadic and scattered tissue positivity was detected in the acute group, even if not in all cases, with the exception of newborns belonging to the non-acute group in which the expression was positive in all cases.

Regarding apoptosis, we know that the damage caused by hemorrhagic lesions is primarily destructive with secondary ischemic alterations. For example, in germinal matrix cells, in presence of hemorrhagic lesions, apoptosis must be interpreted as a secondary phenomenon. Also, there is scientific evidence that ponto-subicular necrosis in cases of perinatal hypoxic-ischemic brain damage is partly to be related to cellular oxidative stress with typical apoptotic alterations with DNA cleavage [9].

The results about the tested antibodies, the semi-quantitative evaluation and statistical analysis are summarized in Table 2 and Table 3.

**Table 2:** Results of immunohistochemical markers response in glial cells

	Acute (A) (≤8 hs) Median Expression	Non-acute (NA) (≥8 hs ≤48 hs) Median Expression	Controls (C) Median Expression	Significant Significance Level Levels	
**GFAP**	+++	+++	+++	A vs C	ns
				NA vs C	ns
				A vs NA	ns

**TNF-α**	-	+	-	A vs C	***
				NA vs C	ns
				A vs NA	***

**IL-1β**	-	+	-	A vs C	***
				NA vs C	ns
				A vs NA	***

**IL-6**	-	++	-	A vs C	***
				NA vs C	ns
				A vs NA	***

**MAC387**	++	+	-	A vs C	***
				NA vs C	***
				A vs NA	**

**HSP 27**	-	-	-	A vs C	ns
				NA vs C	ns
				A vs NA	ns

**HSP 70**	+	++	-	A vs C	***
				NA vs C	***
				A vs NA	***

**HSP 90**	+	++	-	A vs C	***
				NA vs C	***
				A vs NA	***

**COX2**		++	-	A vs C	***
				NA vs C	ns
				A vs NA	***

**ORP-150**	-	++	-	A vs C	***
				NA vs C	ns
				A vs NA	***

**β-APP**	+	++	++	A vs C	***
				NA vs C	***
				A vs NA	ns

**TrypH**	-	-	-	A vs C	ns
				NA vs C	ns
				A vs NA	ns

**GAP-43**	-	+	-	A vs C	***
				NA vs C	ns
				A vs NA	***

**Apoptosis (TUNEL)**	+	++	-	A vs C	***
				NA vs C	***
				A vs NA	***

The reactions were graded as follows: 1. (0): not expressed, 2. (+): isolated and disseminated expression, 3. (++): expression in groups or widespread foci, 4. (+++): widespread expression.

NS = p > 0.05, * = p < 0.05, ** = p < 0.01, *** = p < 0.001

**Table 3:** Results of immunohistochemical markers response in neurons

	Acute (A) (≤8 hs) Median Expression	Non-acute (NA) (≥8 hs ≤48 hs) Median Expression	Controls (C) Median Expression	Significance Level	
**GFAP**	-	-		A vs C	ns
				NA vs C	ns
				A vs NA	ns

**TNF-α**	-	+	-	A vs C	***
				NA vs C	ns
				A vs NA	***

**IL-1β**	+	++	-	A vs C	***
				NA vs C	***
				A vs NA	***

**IL-6**	-	+	-	A vs C	***
				NA vs C	ns
				A vs NA	***

**MAC387**	-	-	-	A vs C	ns
				NA vs C	ns
				A vs NA	ns

**HSP 27**	-	-	-	A vs C	ns
				NA vs C	ns
				A vs NA	ns

**HSP 70**	+	+++	-	A vs C	***
				NA vs C	**
				A vs NA	***

**HSP 90**	+	+++	-	A vs C	***
				NA vs C	**
				A vs NA	***

**COX2**	+++	+	-	A vs C	***
				NA vs C	***
				A vs NA	**

**ORP-150**	++	+	-	A vs C	***
				NA vs C	***
				A vs NA	**

**β-APP**	+	+++	++	A vs C	***
				NA vs C	**
				A vs NA	**

**TrypH**	-	+	-	A vs C	***
				NA vs C	ns
				A vs NA	***

**GAP-43**	-	-	-	A vs C	ns
				NA vs C	ns
				A vs NA	ns

**Apoptosis (TUNEL)**	+	++	-	A vs C	***
				NA vs C	**
				A vs NA	***

The reactions were graded as follows: 1. (0): not expressed, 2. (+): isolated and disseminated expression, 3. (++): expression in groups or widespread foci, 4. (+++): widespread expression.

NS = p > 0.05, * = p < 0.05, ** = p < 0.01, *** = p < 0.001

## Discussion

The immunohistochemical picture obtained using the panel of the antibodies, has shown the existence of a rather precise chronology of expression of the different markers of hypoxic-ischemic brain damage in newborns, which is correlated to the duration of the same insult and is to be ascribed, essentially, to a different stimulation of the different cellular types and also to a different response by the same cells to the ischemic insult. Some immunohistochemical markers have been shown to be more reliable than others in the evaluation of the timing of the neonatal hypoxic-ischemic damage. In particular, chaperonins HSPs have provided very interesting results [10]. Recently, has been demonstrated that brain ischemia depletes ATP and changes intracellular homeostasis, thus disabling ATP-dependent protein quality control systems including molecular chaperones, folding enzymes, and protein degradation components during and after ischemia [10]. In our study HSP70, which is not expressed in control cases, has shown a mild positivity in both neuronal and glial scattered foci beside a weak intravascular positivity, since the acute stages of the hypoxic insult. In the non-acute group, a greater neuronal and glial involvement and intravascular positivity of HSP70 expression has been observed. The reaction was more intense in the cytoplasm of the neurons and included infrequent HSP70 protein positive inclusions in glial cells of the non-acute group [11]. HSP90, characterised by later expression, showed its highest expression in non-acute cases at neuronal, glial and intravascular level, compared with the acute group.

Data emerging from the reaction with ORP150 are in agreement with those reported in literature [12], showing a constant neuronal and glial positivity, in scattered but widespread foci, in most cases belonging to the non-acute group and being completely negative in the glial cells of the acute group. ORP150 was acutely expressed in neurons, strongly suggesting that strongly expression of ORP150 may confer neuronal resistance to early ischemic injury [12]. Therefore, this antigen-antibody reaction can provide useful indications about the time of onset of the hypoxic-ischemic damage.

COX2, the protein responsible of prostaglandin synthesis [13], expressed by vascular endothelium in control cases, appeared more positive not only at vascular level but also in glial cells in non-acute cases. In contrast, COX2 was strongly expressed in neurons in the acute group. This finding will need further development because it contrasts with data reported in literature in which is described a greater expression in the acute cases of asphyxia in the absence of a precise time reference [14].

Our results demonstrated that molecular chaperones HSP70 and HSP90 are strongly expressed in neurons as later reaction [15]. We have demonstrated that ORP150 expression is correlated with cell survival indicating that induction of this stress protein may confer neuronal resistance to acute ischemic injury [12]. COX2 is a precious marker to detect acute neuronal damage during the early stage of focal ischemic encephalopathy [13].

The international scientific panorama is actually rich of studies aiming to the research of histological, histochemical and immunohistochemical markers which can provide more and more accurate information about the time of onset of hypoxic-ischemic brain damage. However, data reported by literature appear fragmentary and contradictory, often concerning experimental studies performed only on animals. Moreover it should be underlined that most reports concern experimental studies which have used markers with late expression (>24 hours) which, therefore, are scarcely reliable in cases of perinatal hypoxic-ischemic brain damage due to intra-partum asphyxia. These neurobiological insights into the mechanisms of the cellular responses implicated in perinatal brain damages, and the characterization of the various mechanisms involved might open new horizons for understanding the time of onset of a brain hypoxic-ischemic lesion and for effective therapeutic strategies [16]. It is well known that the ability of current technologies, such as electronic foetal monitoring, to identify intrapartum hypoxia-ischemia is limited [17]. Neuroradiological techniques to identify injury in the newborns cannot resolve the timing of the injury to within several hours without considerable input from the clinical history (abruption, prolapse, rupture, etc.) or the CTG tracing, but they can apparently exclude an ante-partum process. In cases of neonatal death it is essential the contribution of post-mortem examination complemented by toxicological, microbiological and genetic investigations [18]. The histological study of the brain with traditional histochemical techniques can provide relevant data since it is well known that depending on mechanism, severity and timing of the insult, the distribution and the histological pattern of lesions in the brain changes dramatically [5,19]. By means of immunohistochemical techniques applied in studies both on animals and humans, it has been possible to identify in the brain tissue some markers of hypoxic-ischemic damage with reliable and reproducible results [20-23].

In particular, chaperonins HSP70 and HSP90, ORP150 reaction, and COX2 protein, have provided very interesting results. Finally, these results would suggest a message to the clinicians to extend the differential diagnosis of a too large neonatal hypoxic-ischemic insult chronological category to delineate a more accurate judgement [16].

## Competing interests

The authors declare that they have no competing interests.

## Authors' contributions

IR performed the confocal microscopy analysis. MN carried out the immunohistochemical analysis. FDS drafted the manuscript. CP, EF and FV performed the microscopic analysis. RR participated in the design of the study and performed the statistical analysis. ET and VF conceived of the study, and participated in its design and coordination and helped to draft the manuscript.

All authors read and approved the final manuscript.
